# A systematic mapping literature review of education around sexual and gender diversities

**DOI:** 10.3389/fsoc.2022.946683

**Published:** 2022-08-23

**Authors:** Christian Fernando López-Orozco, Edgar Omar López-Caudana, Pedro Ponce

**Affiliations:** ^1^Institute for the Future of Education, Tecnologico de Monterrey, Mexico City, Mexico; ^2^School of Engineering and Sciences, Tecnologico de Monterrey, Mexico City, Mexico

**Keywords:** educational innovation, sexual and gender diversity, higher education, systematic review, LGBTQ+ community

## Abstract

Education around sexual and gender identities is highly important to understand diversity and prevent discrimination, violence, and even murder. Nevertheless, educational institutions around the world are lacking a curriculum that explicitly includes diversity and acknowledges the LGBTQ+ community, a minority that over the years has been facing consequences from this exclusion. This study presents a detailed description of the process applied to analyze the studies using a systematic mapping literature review, as well as the positive results found from those educational institutions that started their path to inclusion around sexual and gender diversities through their curricula. The research questions targeted in this work are: What is being taught in educational institutions regarding sexual and gender diversities? What are the approaches used inside the classrooms to teach sexual and gender diversities? Which students are receiving education regarding sexual and gender diversities? Is there a technological approach and/or tool used to teach sexual and gender diversities? After applying the filtering processes, 69 studies were selected from five different online libraries: ACM, DOAJ, Lens.org, SCOPUS, and SpringerLink. The conclusions made from the findings of this review are that those studies that do tackle concerns around the topic have proven to benefit the LGBTQ+ community, the education around sexual and gender diversities predominates within the healthcare field, there are a lack of studies around this topic in Latin American countries, and technological tools are minimally used during the teaching processes.

## Introduction

Understanding the differences that make up our society is highly important to acknowledge people's rights and contribute to their development as members of the society. These distinctions make every society unique, but in some cases, they can also represent a challenge when the understanding of their differences is lacking or nonexistent.

Educational institutions play a significant role in the development of a society and its members. Therefore, they have an added responsibility of not only educating but also creating safe spaces in which everyone can express themselves. The globalized and changing world we live in requires constant adaptation, where differences in skin colors, gender, sexual orientation, etc., are more visible every day. Hence, the teaching of sexual and gender diversities within educational institutions is one of those important factors as it is necessary to make this matter visible and thus provide safe spaces for the members of the LGBTQ+ community. However, teaching methods and school staff may continue to be rooted in traditional and patriarchal beliefs, resulting in the exclusion of diversity issues, and further leading to different forms of violence or intolerance.

This systematic mapping literature review attempts to analyze those institutional efforts around the world in which the content regarding sexual and gender diversities has been included in the curricula. Many organizations have been expressing the need for inclusion within educational institutions, and such is the case of the United Nations with its fourth Sustainable Development Goal (SDG) pointing to the quality of education, with the creation of target 4.a that aims to build and upgrade education facilities that are child, disability, and gender sensitive and provide safe, non-violent, inclusive, and effective learning environments for all (United Nations, 2016). Additionally, the OUT IN THE OPEN report published in 2016 by UNESCO (2016) provides the list of suggestions below in order to follow effective responses to school-related homophobic and transphobic violence:

- Monitor systematically the prevalence of violence in educational settings, including violence based on sexual orientation and gender identity/expression.
- Establish comprehensive national and school policies to prevent and address violence in educational settings, including violence based on sexual orientation and gender identity/ expression.
- Ensure that curricula and learning materials are included.
- Provide training and support to teachers and other education and school staff to prevent and address violence in educational settings, including violence based on sexual orientation and gender identity/expression.
- Ensure safe school environments are inclusive and provide support for students affected by violence, including violence based on sexual orientation and gender identity/expression and their families.
- Provide access to nonjudgmental and accurate information on sexual orientation and gender identity/expression through information campaigns and partnerships with civil society and the wider school community.
- Evaluate the efficiency, effectiveness, and impact of education sector responses to violence, including violence based on sexual orientation and gender identity/expression.

When it comes to explicit changes in the curricula, the aforementioned report also expresses the lack of commitment from countries around the world. Only a few countries have developed curricula that explicitly include sexual and gender diversities, and even though countries have policies that mandate including them as part of the educational materials, they are rarely implemented.

To summarize, institutional efforts must include the preparation of issues beyond instructional methodology to teachers and administrators and adaptation of the curriculum and school management and infuse more courses related to educational equity (Kahn and Gorski, 2016). Considering the current literature around school practices, this study attempts to find published works in which some or most of these guidelines are being followed. This leads to the study of the impact and the status of education around sexual and gender diversities.

The focus of this report is then to find those studies in the existing literature in which the efforts toward the inclusion of sexual and gender diversities have reached the curricula. To do the analysis, every educational institution was considered regarding their field or educational level, so comparisons can be developed between those sectors in which the topic is addressed the most. Additionally, part of the study includes not only a review on the use of technology during the teaching processes but also the study to exclusive works related to STEM education.

Finally, this study makes the following contributions regarding education around sexual and gender diversities:

- Understanding the current practices of educational institutions around the inclusion of sexual and gender diversities in their contents.
- Identifying the intervention of technology around the topic.
- Outlining the areas of opportunity for those external sectors not mentioned in the review.

## Mapping process

This systematic mapping process (SMP), based on the current information in the online libraries and the guidelines for systematic mapping studies suggested by a previous study (Kitchenham and Charters, 2007), has the aim of analyzing the existing studies related to the teaching of sexual and gender diversities in educational institutions around the world.

To achieve the goal, several steps are defined that could lead to those studies that comply with the necessary contents established, starting with the definition of the research questions (RQs). Once the RQs are defined, the next step is to develop the search string with those keywords that would allow the study to find higher number of studies online, followed by the definition of the online libraries where the search string will be introduced. Then, the selection criteria must be defined as a first filtering process that could be placed online, with some considerations to include or exclude studies such as year of publication and type of articles. A second filtering process then must be placed, with guidelines of quality evaluating the remaining works. Before the analysis and classification of the final works, which is the final step, a snowballing process will be considered for this study to possibly include works that were not found in the online libraries but that could be found through the references of the ones being considered. In summary, the steps to follow in the SMP are the following:

1) Define the RQs
2) Develop the search string
3) Select online libraries
4) First filtering stage: online
5) Second filtering stage: inspection
6) Snowballing
7) Analysis and classification

### Research questions

The goal of this review is to understand how inclusive curricula toward LGBTQ+ topics are being taken into consideration inside educational institutions around the world, and to understand the dynamics around the topic, the following research questions (RQs) were selected:

RQ1. What is being thought in educational institutions regarding sexual and gender diversities?

RQ2. What are the teaching approaches used inside the classrooms to teach sexual and gender diversities?

RQ3. Which is the main public receiving education regarding sexual and gender diversities?

RQ4. Is there any technological approach and/or tool used to teach sexual and gender diversities?

### Search string

The search string was designed to answer the RQs in the best way possible, focusing on those terms that would lead to the articles that could possibly fit in the study. The terms selected were as follows:

- *School—*as the place where the research is intended to be taken place. This term was considered as this review focuses on the methodologies and contents inside a classroom, looking to exclude those works that happen outside educational institutions.
- *Education—*as the context inside the school. This term was selected as part of the string because of its relevance within research studies, looking for a higher amount of studies because of the many educational fields around academia.
- *Teaching—*as the way education is being transmitted. There is some content being exchanged or taught from a professional to the students.
- *LGBT*—as the topic being taught. They are many ways to refer to the LGBTQ+ community and none of them are wrong since there is not an official one to refer to it, but for the study, the “LGBT” was picked because it encapsulates most of the terms existing and it could reach a higher number of studies.

Synonyms of each term, such as college, schooling, pedagogy, and LGBTQ+, were considered during the online search in the libraries, ending up in results with similar outcomes. It was decided for the simplicity of this study to keep just the search string with the four original keywords, as it would not be a major difference with the data obtained with a longer search string.

### Source selection

To enrich the study and give it a wider point of view, five different online libraries were considered to carry on the search. These five libraries were picked because of their relevance in the topics related to STEM education and its connection with gender studies, as well as their reliability and value of information with a wide range of journals and conference papers, which each one of them contains. The five chosen libraries are as follows:

- ACM
- DOAJ
- Lens.org
- SCOPUS
- SpringerLink

### Inclusion and exclusion criteria

Once the research questions were placed, the search string was defined and the online libraries were selected, and the next step was to create the inclusion and exclusion criteria. This first filtering process aimed to use the online tools of each library to select those papers that comply with the following points:

The inclusion criteria are as follows:

- The paper is written in English or Spanish.
- The work was published after the year 2005.
- The paper was published in a peer-review journal or conference.
- The work includes at least two of the terms related to the topics of the research questions.
- The work develops inside an educational institution.

The exclusion criteria are as follows:

- Studies that are not papers
- Studies not published in a peer-review journal or conference
- Studies that do not answer any of the research questions
- Studies that cover the work but just in a theoretical way

### Quality assessment

A second filtering process after the inclusion and exclusion criteria was carried out online. A quality index was considered with 10 questions that would help the study determine if the paper would provide enough information to answer the RQs. The checklist for quality assessment (Chks) was developed following the guidelines published by the CRD (CRD, 2008), in which 11 points are placed to verify that the systematic review would include enough information to continue the study. These are the resulting questions readapted to suit the target of the study:

- Chk1. Does the work include a real application inside an educational institution?
- Chk2. Does the work describe the methodological approach used to reach the target group?
- Chk3. Are the aims of the work properly described?
- Chk4. Does the work explicitly describe the research context?
- Chk5. Is all the analysis properly supported by literature?
- Chk6. Does the work intend to support the LGBT+ community?
- Chk7. Does the work explicitly mention the target population?
- Chk8. Is the teaching material based on official literature or supported by organizations?
- Chk9. Are the results and findings properly analyzed?
- Chk10. Is the work based on research?

All the checklist questions can be answered with “Yes,” “Partially,” or “No,” and each answer was assigned a specific score (1, 0.5, or 0, respectively) to evaluate the quality of each paper. This evaluation was carried out with an inspection procedure by reading the title and abstract of each paper. In case they did not provide enough information to answer one of the questions, before assigning a 0 (“No” answer), a quick read through the body of the paper was conducted. Also, while filling the checklist and analyzing the database, some patterns were found in similar works that allowed the process to be more efficient such as checking the keywords or just checking the subtitles to discern if the missing questions could be answered or not.

All those studies whose score was higher than 7 were included in the following sections. This number was chosen after an iterative process of comparisons between the quality of studies. Multiple studies were compared regarding their contributions to the topic and classified accordingly with the final scores derived from the checklists, concluding that those studies with scores lower than 8 points were lacking most of the information needed to respond to more than one of the mapping questions.

### Snowballing

The snowballing process was applied using the results of the remaining articles after all the filtering processes as well as excluding some of the studies that instead of conducting an original work were papers showing reviews of different papers that do fill the quality to be included in this study.

Some of the papers that did not fulfill the quality assessment were articles making comparisons between the quality of multiple works, so they were an important resource to find those that would fulfill the quality assessment and that were not found in the chosen online libraries. From the remaining works, the author would go to the reference section of each of the articles and would first review the title of the research and then apply the full checklist to make sure it was applicable for contributing to the study after finding the complete work.

## Data gathering

After all the filtering processes were done, some mapping questions (MQs) were placed to analyze the papers selected. These questions have the aim of extracting relevant information for the research intended, which could provide extra information to the study and help the RQs to have a wider and more detailed answer.

- MQ1. Which are the main educational institutions in which topics regarding sexual and gender diversities are being taught or studied?
- MQ2. How can the studies be classified according to the educational context?
- MQ3. Which are the most relevant journals, authors, and countries carrying out studies regarding teaching sexual and gender diversities?
- MQ4. Which years are the most prevalent in which works around sexual and gender diversities have been published?
- MQ5. Which are the main methodologies used inside educational institutions to gather data from studies around sexual and gender diversities?

**Figure 1:**
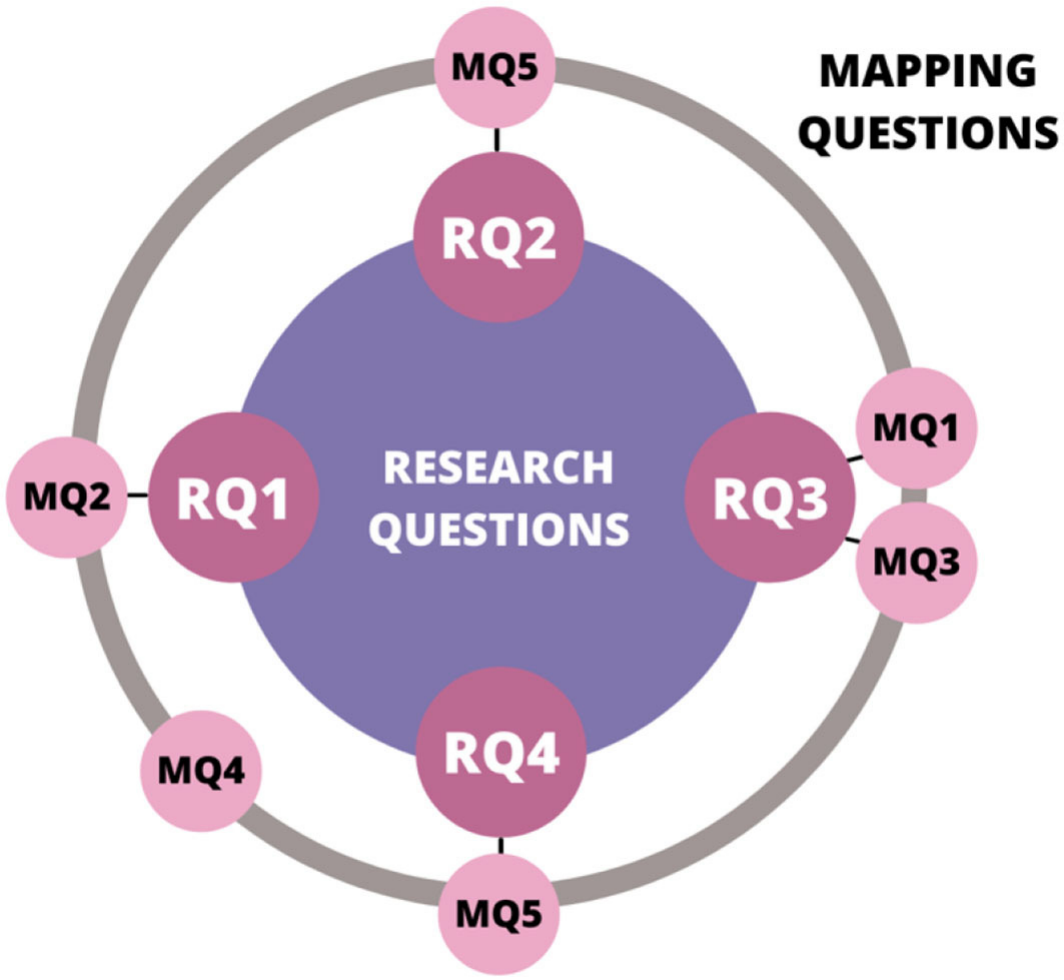
Relation between RQs and Qs.

Figure 1 shows how the MQs contribute to the RQs. It can also be observed that MQ4 does not have a direct relation to any of the RQs, but it gives important information about the dynamics of the publications over time.

Table 1 shows the structure used to extract the information, that is, the item or title of each section, the description of which information to place in the database, and how each item relates to either the RQs directly or indirectly through the MQs.

**Table 1:** Structure of the data extracted.

**Item**	**Description**	**Qs and RQs that relate**
Title	Title of the publication	
Publication year	Year of publication	MQ4
Publication type	Publication type (journal article, article, conference paper, or book chapter)	
Source title	Journal or book name	MQ3
Publisher	Name of the publisher	MQ3
Source country	Country	MQ3
Institution	Type of institution (primary, high school, university, etc.)	MQ1
Author/s	Name of all authors	MQ3
Abstract	Abstract	
Keywords	All keywords	RQ1
DOI	DOI	
Library	Online library (AMC, DOAJ, Lens, Scopus, Springer, or Snowballing)	
Target group	Target population	RQ3
Topic	Main topic of the article	MQ2
Field	Main field of work	MQ2
Methodology	Methodology used to teach or study the target population	RQ2 and MQ5
Content	What is being taught or studied?	RQ1
Tech-tools	Technological methodology used	RQ4

All the information was structured in a database to have it normalized and make the analysis easier.

## Data analysis

This section shows the data before and after the filtering processes were applied using quantitative analysis. At the end of the section, all the MQs will be answered and analyzed using the studies that comply with every filtering stage.

Table 2 shows the search string used in each online library and the total amount of studies without filtering. A total of 4,278 articles were considered at the initial stage, of which more than half of them were found in the SpringerLink library.

**Table 2:** Online search and results.

**Online library**	**Search string**	**Result**
ACM	(((“LGBT”) AND (“School”) AND (“Education”) AND (“Teaching”)))	43
DOAJ	Education AND LGBT AND Schools AND Teaching	28
Lens	Education AND (LGBT AND (School AND Teaching))	858
SCOPUS	Education AND LGBT AND School AND Teaching	408
SpringerLink	Education AND LGBT AND Schools AND Teaching	2,941

**Figure 2:**
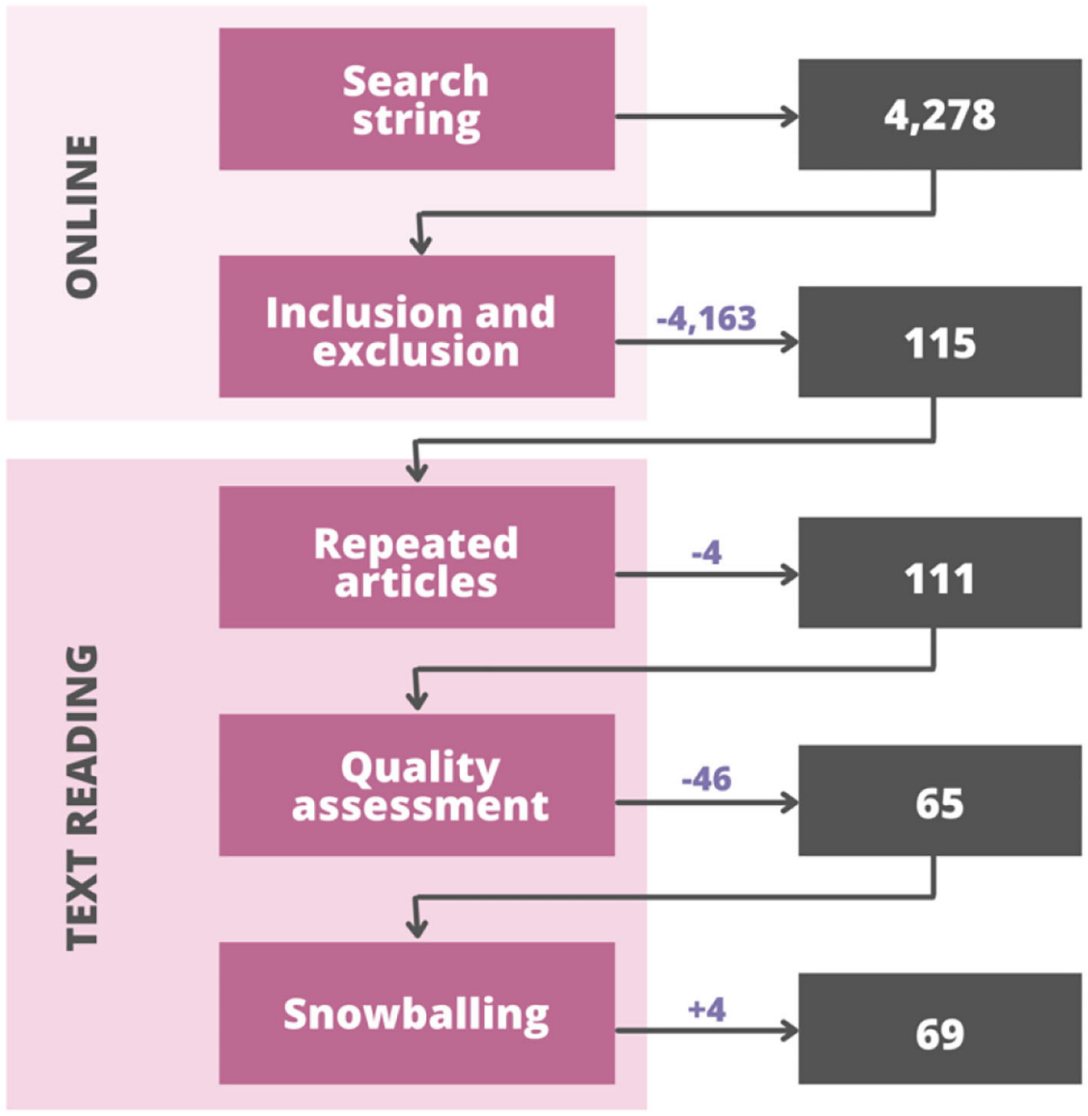
Selection process and total of articles.

The general view of all the selection processes is shown in Figure 2, which starts with the total amount of studies found in the search string. After applying the online filtering process, 4,163 works did not meet the requirements of the inclusion and exclusion criteria, so a total of 115 studies (2.69% of the initial number) were used for the text reading section. Before applying the quality assessment, the database was checked to identify those works that could have been found in more than one online library, and a total of four were identified and thus removed from the total amount to avoid double-checking them during the quality assessment. The remaining 111 studies entered the quality assessment and 46 did not meet the criteria; therefore, they were also removed from the database. Finally, the snowballing procedure was applied of which four works were found that met the necessary requirements and were thus added to the database. This procedure consisted of using the remaining works in addition to some of the previously eliminated in order to check for published works that were eligible for the study but were not originally found in the online libraries. A total of 69 works were then considered for the study, which represents 1.61% of the total amount of works found at the initial stage in the search string.

The final distribution of the works per library is shown in Table 3, with the number of studies before and after applying all the filtering processes shown in the columns “Number initial stage” and “Number final stage,” respectively. Also, in the “Studies found” column, the identifier of each work for the reference section of this paper is shown.

**Table 3:** Total of studies selected.

**Library**	**Number initial stage**	**Number final stage**	**References**
ACM	43	2	Acena and Freeman, 2021; Chuanromanee and Metoyer, 2021
DOAJ	28	12	Epstein et al., 2013; Bakhai et al., 2016; Francis and Reygan, 2016; Underman et al., 2016; Cooper et al., 2018; Dudar et al., 2018; Santos et al., 2018; Geunis and Holz, 2020; Nowaskie and Patel, 2020; Acena and Freeman, 2021; Minturn et al., 2021; Tollemache et al., 2021
Lens	858	23	Ellis, 2009; Jackson, 2010; Røthing and Svendsen, 2010; Sauntson and Simpson, 2011; Bacon, 2013; Neary, 2013; Riggs, 2013; Riggs and Due, 2013; Tang, 2014; Formby, 2015, 2017; Duque Sanchez and Teixido, 2016; Flores, 2016; Falconer and Taylor, 2017; Hope and Hall, 2018; Slater et al., 2018; Henderson, 2019; Lee, 2019; Taylor and Cuthbert, 2019; Wood et al., 2019; Gelís and Morales, 2020; Grant et al., 2021; Llewellyn and Reynolds, 2021
SCOPUS	408	18	Eliason et al., 2011; Schmidt et al., 2012; Mandap et al., 2014; Seaborne et al., 2015; Aaberg, 2016; Helmsing, 2016; Magnus and Lundin, 2016; Bonvicini, 2017; Bristol et al., 2018; Hickerson et al., 2018; Johnson et al., 2018; Higgins et al., 2019; McDowell et al., 2020; McEwing, 2020; Nowaskie and Patel, 2021; Ray King et al., 2021; Sherman et al., 2021a,b
SpringerLink	2,941	10	Knotts, 2009; Broadway, 2011; Harris, 2013; Muntinga et al., 2016; Holthouser et al., 2017; Beck Dallaghan et al., 2018; Sinacore et al., 2019; Altneu et al., 2020; Ho, 2020; Pratt-Chapman, 2020
SNOWBALLING	0	4	Bennett and Reddy, 2007; Eckstrand et al., 2017; Sanchez et al., 2017; Ufomata et al., 2018
TOTAL	4,278	69	

It is important to notice that, even though the SpringerLink library had a significantly higher amount of works at the initial stage, it did not end up having the highest number of studies considered for the final stage. The DOAJ online library had the lowest percentage of reduction, with a total of 42.86% of studies included for the final stage compared to its initial number found during the search string.

Sixty-seven of the final works selected at the end are published papers or journal articles, but this study also included two extra publications because of their relevance to the topic: a book chapter (Acena and Freeman, 2021) and a conference paper (Chuanromanee and Metoyer, 2021).

The MQs were now answered considering only the remaining 69 works that met the filtering processes, the inclusion and exclusion criteria, the quality assessment, and the snowballing procedure.

### MQ1. Which are the main educational institutions in which topics regarding sexual and gender diversities are being taught or studied?

**Figure 3:**
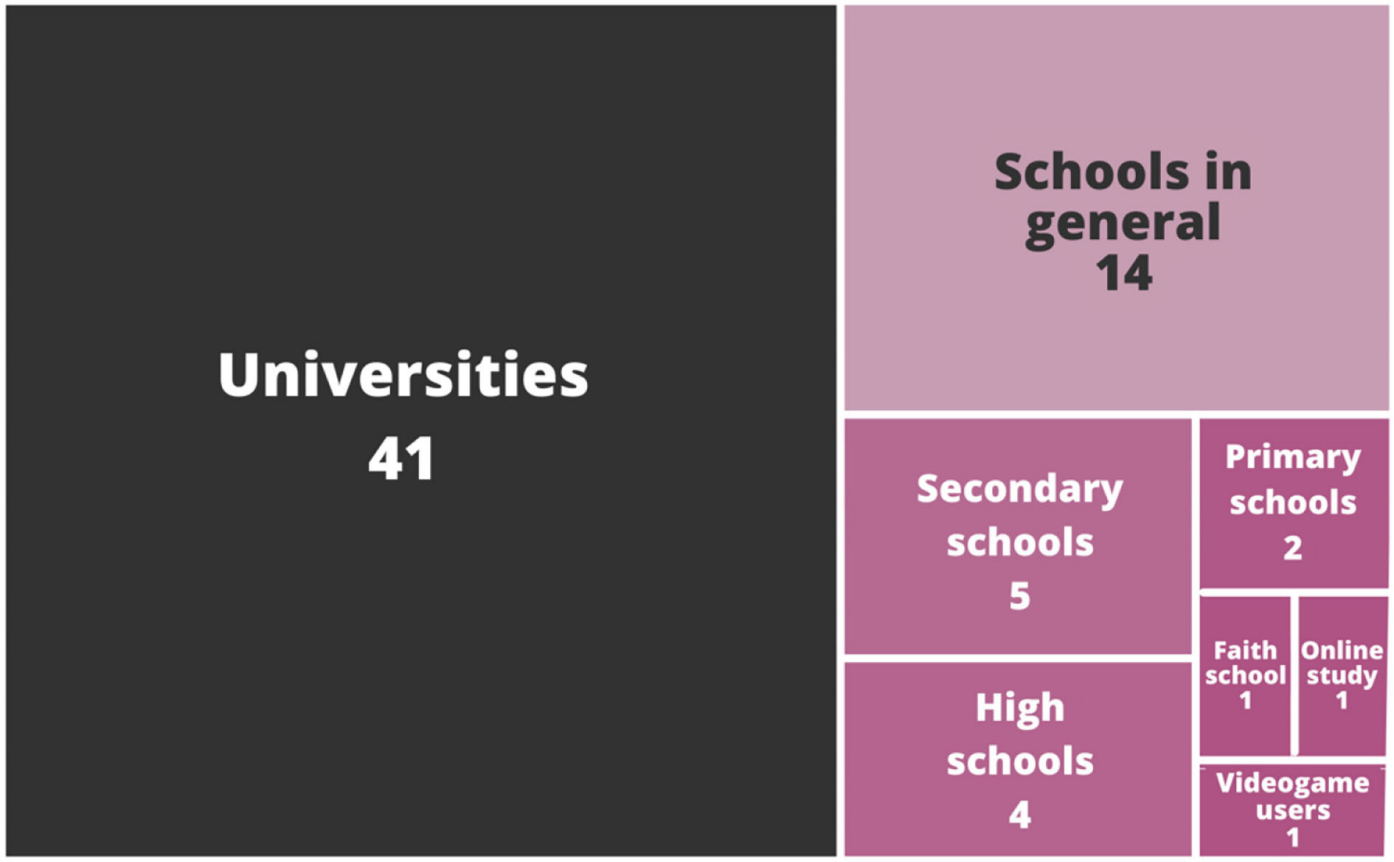
Distribution of institutions.

The general view of the institutions or public found within the selected articles is shown in Figure 3. As observed, the most relevant institutions in which topics regarding sexual and gender diversities are being taught or studied are universities, with 59.24% of the entire studies. The “Schools in general” subsection, 20.28% of the total, stands for those studies that mentioned an educational institution as the target population of the study but did not identify the educational level. The remaining 20.28% of works are institutions of basic education (primary and secondary schools) and high schools, as well as an online study targeting social media users and a study with video game users.

### MQ2. How can the studies be classified according to the educational context?

**Figure 4:**
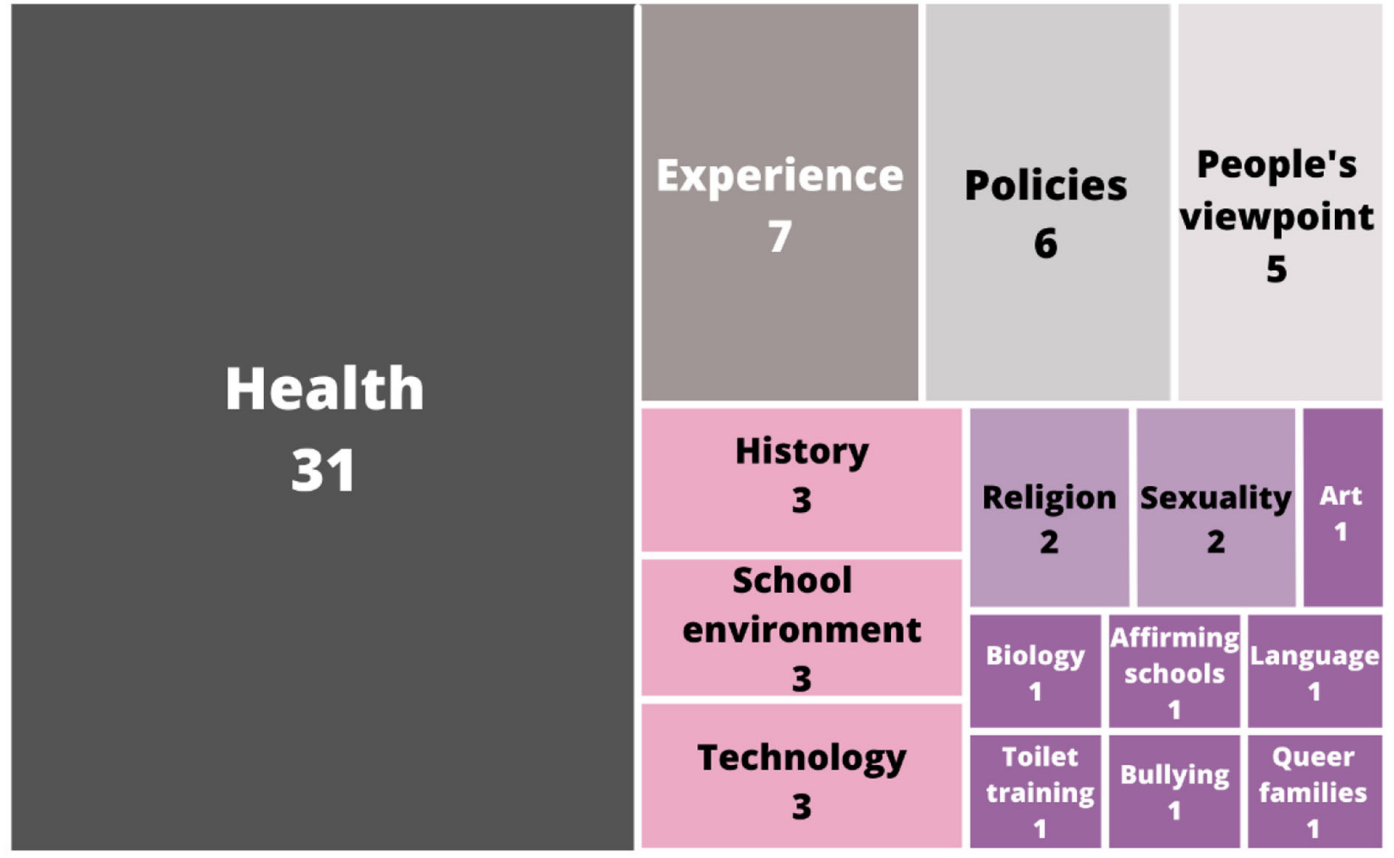
Field of study.

The classification of the educational contexts infers what type of content is being studied around the world regarding sexual and gender diversities. The process of assigning a category of each work was done while carrying out a text reading process, and according to the closest general environment in which it was applied, a total of 16 different categories were found. “Health-” or “Healthcare”-related topics are the most relevant conducting or including education around sexual and gender diversities in their curricula. Some of the relevant studies are centered on people's experiences, perspectives, and the existing policies around them. There are few studies in which diversity is linked to the main subject, such as history or art classes, including a gender perspective, biology, and sexuality classes with a perspective on diversity, and even studies checking on queer identities around religion. Figure 4 shows the general quantitative distribution of the topics.

Three of the works that were analyzed were decided to be placed into their own categories regarding the focus of their studies (toilet training, affirming schools, and queer families). Those outliers were included in the study because they fulfilled the quality assessment, but they could not necessarily be classified in any of the general fields. Therefore, instead of removing them from the study, they were kept and placed in their own field as they would make higher contributions to some of the other mapping questions.

### MQ3. Which are the most relevant countries, journals, and authors carrying out studies regarding teaching sexual and gender diversities?

**Figure 5:**
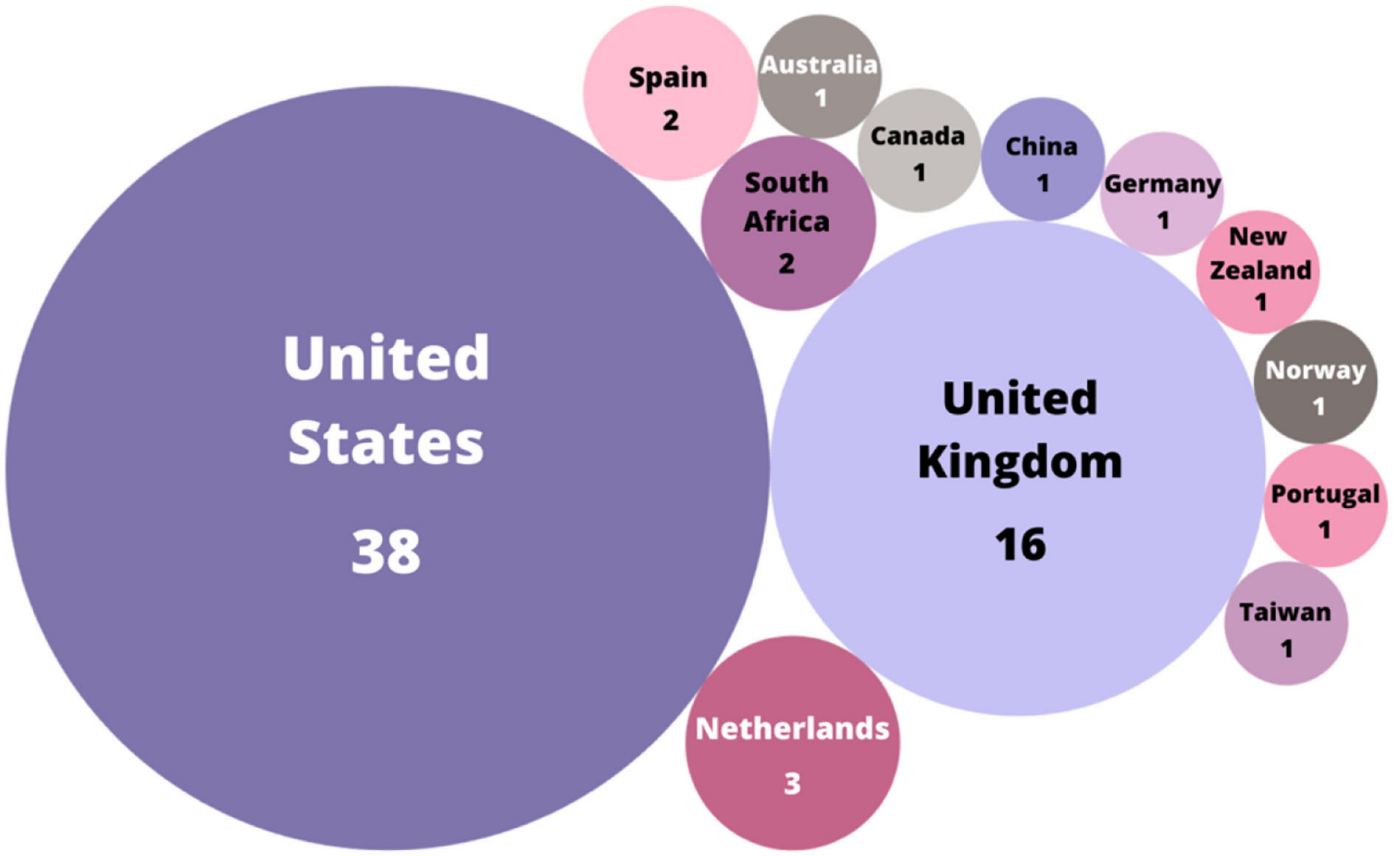
Number of publications per country.

From Figure 5, it can be observed that close to 50% of the total amount of works included in this study is based on the US, meaning that it is the country in which most studies regarding the teaching of sexual and gender diversities are found. The second place for this category belongs to the UK, with 23.19% of the total amount of published works considered. The remaining countries all together add to a total of 15 published works, with eight studies published from Europe, two from Africa, two from Asia, two from Oceania, and one from North America.

The publishing works of top journals regarding the teaching of sexual and gender diversities are all based on the UK and the US. The left side of Table 4 shows the top 10 journals that altogether represent a total of 31 works, with those journals having more than one publication in the database of this study. The remaining 38 works belong to journals with single publications, meaning that the entire collection of journals consists of 48 different titles.

**Table 4:** Top 10 journals and authors.

**Journals**			**Authors**		
**Journal title**	**Country**	**Number of articles**	**Author's name**	**Country**	**Number of articles**
Sex Education	United Kingdom	6	Eckstrand Kristen L	United States	3
MedEdPORTAL	United States	5	Dustin Z. Nowaskie	United States	2
Nurse Education Today	United States	4	Anuj U. Patel		
British Journal of Sociology of Education	United Kingdom	3	Kelly M. Bower	United States	2
BMC Medical Education	United States	3	Kristen D. Clark		
Medical Science Educator	United States	2	Meredith Klepper		
LGBT Health	United States	2	Athena D.F. Sherman		
Journal of Professional Nursing	United States	2	Damien W. Riggs	United Kingdom	2
Journal of Homosexuality	United States	2	Eleanor Formby	United Kingdom	2
Gender and Education	United Kingdom	2	Yvette Taylor	United States	2
Total		31	Total		13

The British journal “Sex Education” leads with publications that discuss the topic, followed by the American journal “MedEdPORTAL.” When it comes to journal titles, it can be observed that the American journals show a pattern around healthcare topics, while each journal in the UK has a different field around education.

Finally, the top 10 authors are shown on the right side of Table 4, with the American author Eckstrand Kristen L writing the most with three of the total amounts of articles considered for the study. Some of the authors that have two publications worked as teams, and the location of the top authors are either in the US or in the UK.

Even though works in English and Spanish were included in the search string of the databases, these results show a vast majority of countries in which the English language is predominant within its population. These results also considered only the top 10 authors and journals in which the number of publications was at least two, so those countries in which only one article was found had no opportunity of appearing in the study, giving the US and the UK a significant advantage by being the countries with the highest amount of articles.

### MQ4. Which years are the most prevalent in which works around sexual and gender diversities have been published?

**Figure 6:**
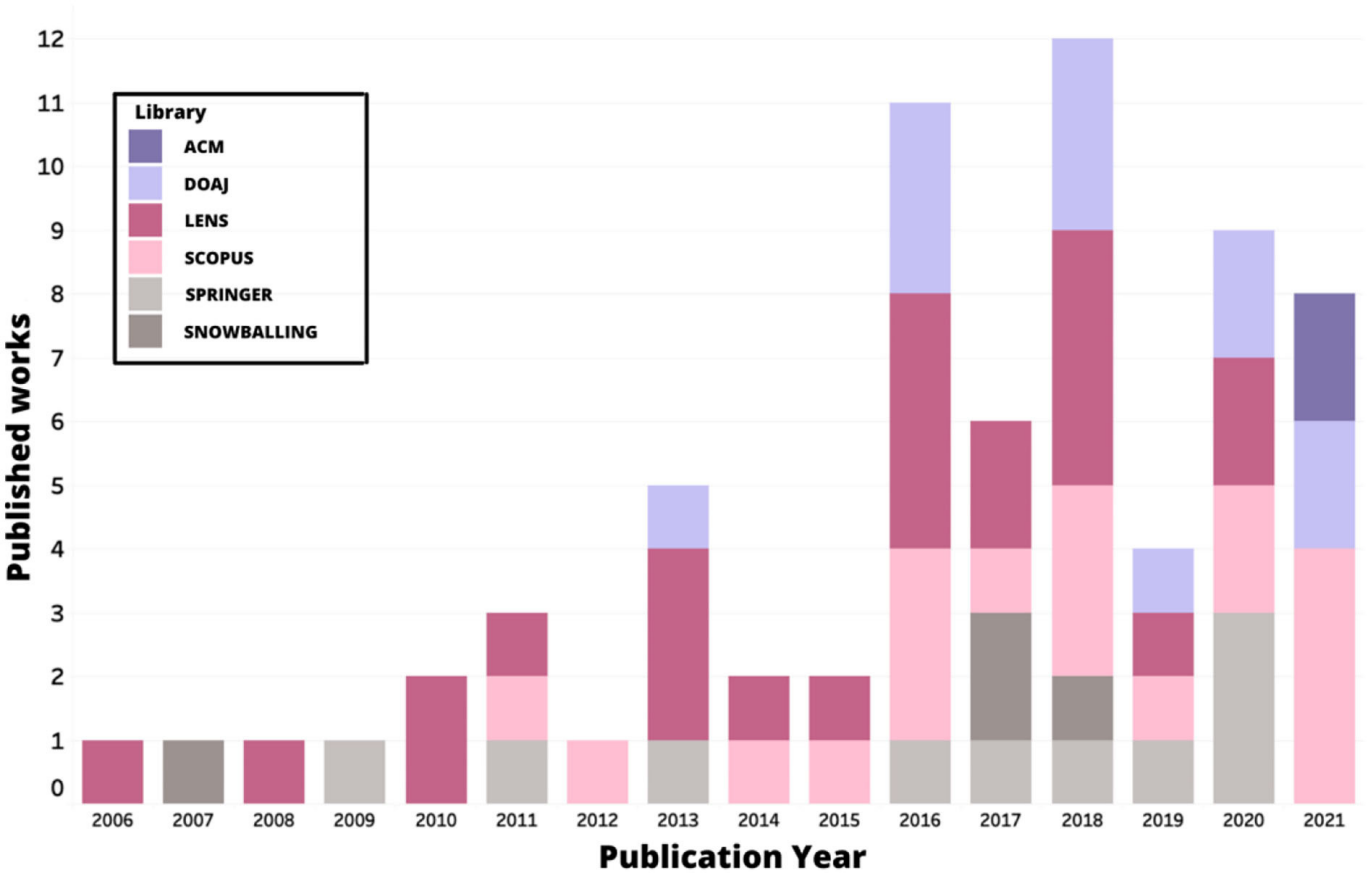
Distribution of publications per year and source.

Figure 6 shows both the evolution of works published around the world from the year 2006 to the year 2021 and the frequency of publications of each online library per year. There has been a notable increment in the number of works after the year 2015, reaching its peak in the year 2018 with a total of 12 works that were considered in this study. It also shows a significant decrease in publications in 2019, which could be attributed to the COVID-19 pandemic and the unforeseen lockdown around the world. With schools closed and the transition to a digital-based education, there could be a cause for the works to be temporarily stopped from publishing. Nevertheless, 2020 and 2021 show a return to the number of publications around the topic, with 9 and 8 works published, respectively.

The Lens and Scopus online libraries show the highest level of consistency in publishing throughout the years, with at least one published paper around the topic for 8 years in a row. Moreover, the ACM online library seems to be the newest when it comes to publications on the topic, with only two publications made during the last year.

### MQ5. Which are the main methodologies used inside educational institutions to gather data from studies around sexual and gender diversities?

**Figure 7:**
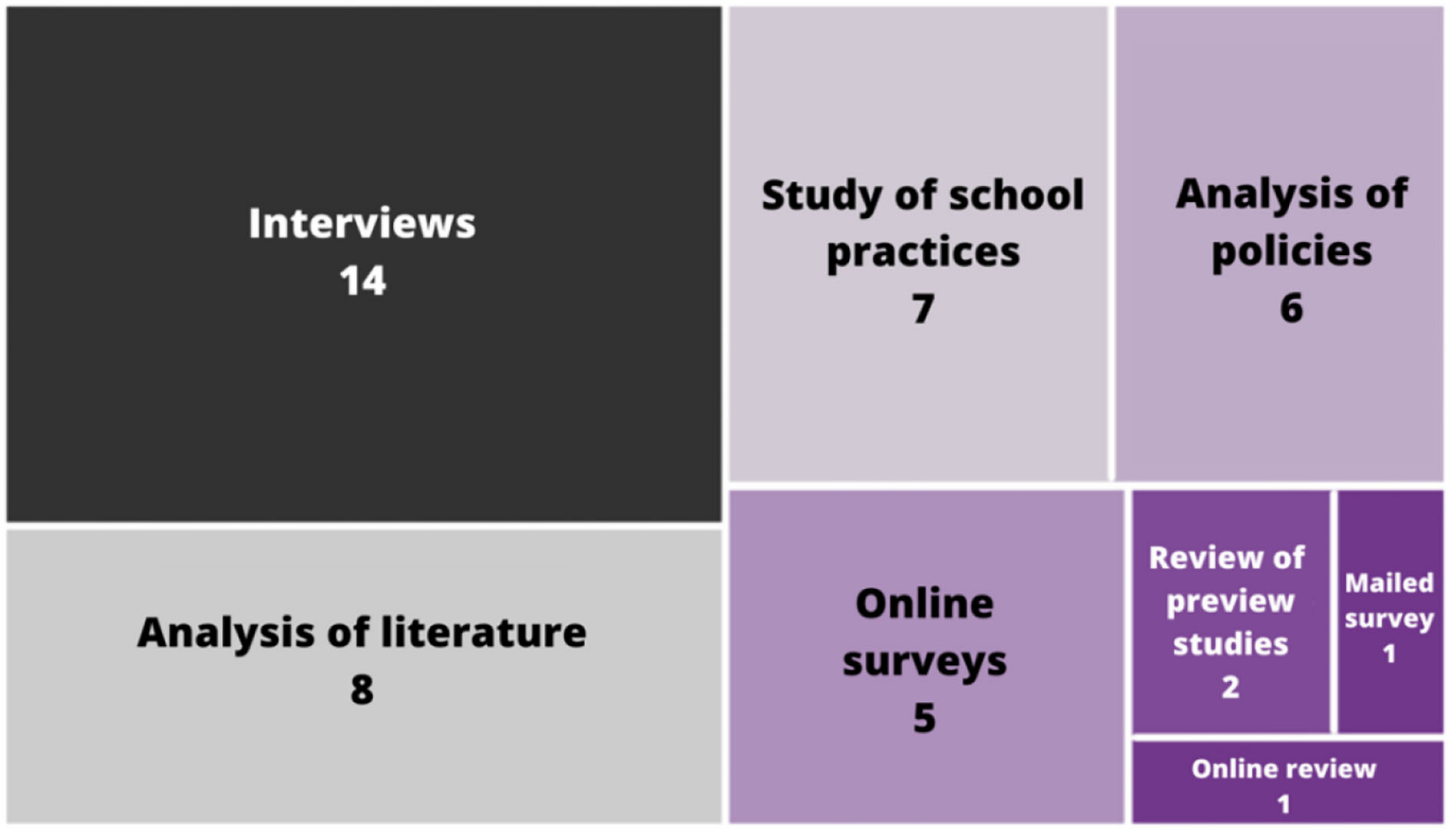
Data gathering.

The main methodologies to gather the data are either through direct interaction with the target population or by reviewing literature from the environments. As seen in Figure 7, from the 44 publications that mentioned their data gathering processes, interviews have been the main tool used to evaluate the studies, followed by the analyses of literature, practices, or policies around people, having their focus not only on giving quantitative measures to their work but also to understanding the spaces. Another few methodologies are online reviews regarding the topic or using other studies that evaluate them and try to find improvements.

## Research outcomes and discussion

This section shows a detailed answer to the RQs based on or supported by the previous analyses made with the MQs. The MQs, as mentioned previously in the study, will help to give a deeper understanding of the RQs by supporting the context of the research. MQ2 supports RQ1 by classifying the topics, MQ5 supports RQ2 by also analyzing the data gathering processes, MQ1 and MQ3 support RQ3 by classifying the contexts, and MQ5 also supports RQ4 by taking those categories in which technology is being used and comparing them to the other works.

### RQ1. What is being taught in educational institutions regarding sexual and gender diversities?

MQ2 already gave a general categorization of the topics being taught inside educational institutions, so to answer RQ1, this will be considered as the starting point by using those classifications, grouping them by similarities of studies and giving them a deeper explanation of the contents presented. The six main topics on which institutions are focused are the following:

- Healthcare
- People's experiences or points of view
- School policies and school environments
- Classes with gender perspectives
- Technology
- Religion

Starting with healthcare, which is the predominant topic being taught, the contents are mostly around patient care and improvements in the curricula to provide better services to people belonging to the community of gender and sexual diversities. Medical, nursing, pharmacy and biomedical students, and staff members are all included in the studies as the main public receiving education around the topic as part of their preparation for the professional field. There are also studies focusing exclusively on analyzing the curricula contents from different schools, and important to identify those institutions that might have room for improvement when it comes to the preparation of their students and providing them with possible frameworks for improvement.

The second category refers to those studies in which the content is being analyzed through people's experiences or points of view of their environments. Students, professors, and LGBTQ+ members share their thoughts as part of the studies, and then, the analysis is developed by the authors. This type of content is necessary within gender studies to understand the environments of the people that experience them daily, so the possible outcomes or suggestions for improvement can come based on the people experiencing the exclusion.

When it comes to school policies and environments, the difference from the previous section is that this does not necessarily include people as part of their data gathering processes. The focus of this type of content is to understand the environment through the study of the school policies and practices being implemented. There are also studies focusing on specific topics such as the inclusion of queer families or even workshops whose purpose is to provide toilet training to tackle normative positions inside institutions. The goal of these studies is also to develop frameworks by comparing other studies so that the institutions can find better practices for the creation of safer spaces for their students.

For the classes with gender perspectives, nine of the studies implemented methodologies in which the main subject of the class was not necessarily diversity, but they managed to find a way to introduce a gender perspective without losing the main target of the class. A study around liberatory pedagogies in art classes showed how they managed to incorporate conversations around diverse sexualities and their findings to tackle discrimination inside the classroom. Also, science classes such as Sexuality and Biology used methodologies to tackle privileges inside the school by introducing topics around the LGBTQ+ community, finding the benefits of introducing these topics to make improvements around their curricula. Three of the studies used History classes to teach and analyze literature around diversity and creating discussions, creating safe spaces, empathy with their peers, and recognizing the works of people belonging to the community of sexual and gender diversities. Finally, there is a study using Content and Language Integrated Learning (CLIL) as a way of developing a critical sexual literacy curriculum that combines gender and sexuality education and critical language teaching, analyzing the emotions toward LGBTQ+ people and their gains around the topic.

The technology field will be described in more detail below in Section RQ4. Is there any technological approach used to teach sexual and gender diversities? as the answer for RQ4. However, those contents being taught around technology include works using medical technology or technological tools centered around people belonging to the community of gender and sexual diversities.

Finally, studies around religion were found, whose aim was to understand the dynamics around queer people or queer religious students, through the understanding of those spaces and their perspective on the topics as well as the school perspective or allowance to diversity.

Table 5 shows the distribution previously described, with the six main topics categorized, their subtopics if applied, and their location in the references section.

**Table 5:** Teaching content.

**Topic**	**Subtopic**	**Studies**	**References**
Health care	Medical students	31	Bakhai et al., 2016; Underman et al., 2016; Bonvicini, 2017; Holthouser et al., 2017; Sanchez et al., 2017; Beck Dallaghan et al., 2018; Cooper et al., 2018; Dudar et al., 2018; Ufomata et al., 2018; Gavzy et al., 2019; Nowaskie and Patel, 2020; Minturn et al., 2021; Tollemache et al., 2021
	Nursing students		Eliason et al., 2011; Aaberg, 2016; Hickerson et al., 2018; McEwing, 2020; Ray King et al., 2021; Sherman et al., 2021b
	Pharmacy students		Mandap et al., 2014; Nowaskie and Patel, 2021
	Biomedical students		Muntinga et al., 2016
	Staff members		Seaborne et al., 2015; Bristol et al., 2018; Higgins et al., 2019
	Transgender care		Johnson et al., 2018; Sherman et al., 2021a
	Framework for improvement		Eckstrand et al., 2017; Altneu et al., 2020
	Contents		McDowell et al., 2020; Pratt-Chapman, 2020
People's experiences or point of views	Students		Bacon, 2013; Formby, 2015, 2017; Magnus and Lundin, 2016; Santos et al., 2018; Geunis and Holz, 2020
	Teachers	13	Neary, 2013; Francis and Reygan, 2016; Henderson, 2019; Lee, 2019; Llewellyn and Reynolds, 2021
	LGBTQ+ community		Sauntson and Simpson, 2011; Tang, 2014
School policies and school environments	Policies	12	Knotts, 2009; Jackson, 2010; Schmidt et al., 2012; Epstein et al., 2013; Sinacore et al., 2019; Gelís and Morales, 2020
	Environment		Ellis, 2009; Røthing and Svendsen, 2010; Grant et al., 2021
	Practices		Riggs and Due, 2013; Hope and Hall, 2018; Slater et al., 2018
Classes with gender perspectives	Art	8	Harris, 2013
	Biology and sexuality		Bennett and Reddy, 2007; Broadway, 2011; Wood et al., 2019
	History		Duque Sanchez and Teixido, 2016; Flores, 2016; Helmsing, 2016
	Languages		Ho, 2020
Technology		3	Riggs, 2013; Acena and Freeman, 2021; Chuanromanee and Metoyer, 2021
Religion		2	Falconer and Taylor, 2017; Taylor and Cuthbert, 2019
Total		69	

### RQ2. What are the teaching approaches used inside the classrooms to teach sexual and gender diversities?

From the total amount of studies, Table 6 shows the 30 works that used a direct way of teaching about gender and sexual diversities and the methodology they implemented. From a general perspective based on the analysis, a traditional lecturing procedure is the main methodology being used inside educational institutions, with the professor sharing the content with a group of students. Workshops are also a common way to teach and share information around the topic, and even though they do not necessarily infer a direct change in the curricula, they represent a way to inform and support people belonging to the community of sexual and gender diversities. The analysis of literature was included as part of the answer because of the frequency of studies that used this methodology to study and understand the context around people. These analyses represent an important tool to understand the environment and to make conclusions based on what type of materials professors use inside the classrooms. Then, focus groups and case studies are the next methodologies on the list, as interesting ways to teach based on the direct interaction with people by solving specific cases or situations and by sharing points of view regarding the topic.

**Table 6:** Articles by teaching approaches.

**Tool**	**Number of articles**	**References**
Lectures	8	Schmidt et al., 2012; Holthouser et al., 2017; Cooper et al., 2018; Dudar et al., 2018; Ufomata et al., 2018; Minturn et al., 2021; Sherman et al., 2021a,b
Workshops	5	Riggs and Due, 2013; Underman et al., 2016; Slater et al., 2018; Gavzy et al., 2019; Ho, 2020
Analysis of literature	4	Formby, 2017; Johnson et al., 2018; Higgins et al., 2019; Ray King et al., 2021
Case of study	3	Helmsing, 2016; Bristol et al., 2018; McDowell et al., 2020
Focus groups	3	Formby, 2015, 2017; Santos et al., 2018
Art class	1	Harris, 2013
Flipped classroom	1	Bakhai et al., 2016
Literature program	1	Flores, 2016
Online modules	1	McEwing, 2020
Seminar	1	Magnus and Lundin, 2016
Simulation program	1	Hickerson et al., 2018
VR platform	1	Acena and Freeman, 2021
Total	30	

Also discovered were some individual studies that included sexual and gender diversities in ways that are worth mentioning in this study, such as the creation of seminars around the topic. Two of the publications found a way to teach classes around art and literature while including content regarding gender and sexual diversities, an interesting way to create safe spaces by challenging and restructuring their contents in the classroom. Three of the publications created off-classroom activities by including flipped classroom techniques, online modules, or simulation programs, leading to a more self-dependent way to learn about the topic. Finally, an approach to the use of virtual reality was identified and thus will be addressed in more detail in Section RQ4. Is there any technological approach used to teach sexual and gender diversities?

### RQ3. Which is the main public receiving education regarding sexual and gender diversities?

As a study centered around educational institutions, students represent the majority of people receiving education as it pertains to the teaching of gender and sexual diversities. In fact, 68.12% of the total amount is represented by students from any educational institution, and if we consider the previous analysis made for MQ1, at least 50% of all those students belong to institutions of higher education.

Teachers as the main public receiving focus when it comes to teaching gender and sexual diversities represent 10.15% of the total amount, on studies in which the professor could either be the receptor of the content or the sharer of the methodologies they use. This second part represents those studies in which the professor challenges the norm by implementing methodologies that match the original goal of the curricula and decides to apply a connection with the topic of diversity, creating a safe space for students and giving room for discussion and empathy.

Finally, the LGBTQ+ community and parents are also two sectors of people included in this study that represent those adults invited from educational institutions to voluntarily participate in studies regarding sexual and gender diversities. There are works carried out by looking at the increase of queer families around education or by just trying to improve their school practices when it comes to diversity. Both sectors are important to analyze, complementing the circle in which students interact daily.

Table 7 shows the distribution and frequency of each target group previously described.

**Table 7:** Target groups.

**Group**	**Number of articles**
Students	47
Teachers	7
LGBTQ+ community	3
Parents	1
Total	58

As it can be noted in the previous table, from the total of 69 works, only 58 specified a direct target population as the focus of the studies, leaving 11 works as outliers for the section.

Of those remaining works, nine put literature as the main subject of study, representing 13.04% of the total publications used for the review. This means that there are studies focusing just on the contents being taught within educational institutions rather than the way of teaching or the people for whom the content is being shared. This type of analysis allows a better understanding of the curricula and allows for improvement in the implementation of better practices. The last two works were those that would solely review the presence of inclusive curricula, analyzing if there were some types of content in which diversity is being included or if it is just being ignored by the institution. Rather than checking on the quality of the content, these last two studies would just make a checklist of the existence or not, without making recommendations or comparisons to achieve inclusion.

### RQ4. Is there any technological approach used to teach sexual and gender diversities?

As the last research question, the aim of RQ4 was to find those connections between gender and sexual diversities with technology, and it was considered at the beginning of the study to be a question that could possibly not have an answer. Nevertheless, Table 8 shows that, out of the 69 publications considered for this study, 13 publications used some technological tool as a part of their procedures, which is 18.84% of the total amount. The main tool used for this topic was data gathering through online surveys compared to those studies that used face-to-face surveys, and the use of a technological tool could facilitate the data gathering process, and its speed could also generate some bias if the people being interviewed do not collaborate properly. The use of social media, both as advertising or as recruitment, represents a modern way to reach people and sectors depending on the type of study that is desired, and it shows that it was part of the processes for four of the publications analyzed in this work. Apps, online modules, videos, and websites all connect to the use of internet platforms in which education can be complemented. Nowadays, the internet, if used properly, could be an extraordinary way to have access to sources of information that were hard to reach in the past, and complementing with specific modules such as applications or websites make it even easier to interact and learn a certain topic.

**Table 8:** Articles using technology.

**Tool**	**Number of articles**	**References**
Online survey	4	Eliason et al., 2011; Aaberg, 2016; Sanchez et al., 2017; Tollemache et al., 2021
Social media advertising	3	Formby, 2015; Taylor and Cuthbert, 2019
Apps	1	Chuanromanee and Metoyer, 2021
Online modules and simulations	1	Hickerson et al., 2018
Social media recruitment	1	Taylor and Cuthbert, 2019
Virtual reality (VR)	1	Acena and Freeman, 2021
Videos	1	Gavzy et al., 2019
Websites	1	Riggs, 2013
Total	13	

Finally, there was only one study that used technology as the main topic of the research by using virtual reality to support and study the community of sexual and gender diversities. This work represents a path to technological inclusion that has been ignored for many years, with a gender perspective centered on users that are looking for safe spaces in which they could be themselves and be recognized.

## Conclusion

As part of the inclusion around educational institutions, the acknowledgment of the gender and sexual diversities within the curricula is fundamental to creating safe spaces for students. The development of content and its integration into the curricula could be a difficult task, but studies around the globe have shown its benefits, both within the educational sector by learning about diversity and toward the LGBTQ+ community by receiving better services. This study aimed to provide a general analysis of the educational sector around gender and sexual diversities, as well as how academia has been focusing on paying attention to the contexts around people belonging to this sector. The results are promising from those few studies that show curricula changes, but it requires a systematic change around the institutions to generate these outcomes.

From the analysis made, it can be noticed that the teaching or studying around sexual and gender diversities is missing from educational contexts outside the healthcare fields. There are a minimum number of studies whose focus is not on any topic within the medical field, which means that there are many sectors in universities that do not acknowledge diversity. There are many professional fields that interact every day with people who identify as part of the LGBTQ+ community, so the preparation of educational institutions has a wide room for improvement when it comes to the preparation of their students to be able to interact and develop a higher understanding of their needs.

Also, those studies in which curricula have been readapted to teach diversities have proved beneficial among the LGBTQ+ community. All those studies in which the curricula have evolved and students are receiving content to improve their services show a promising outcome when it comes to providing services for people who identify it as part of the LGBTQ+ community. Most of the curricula changes, mainly pertaining to the medical field, have shown that professionals have more natural interaction while providing the services to these people compared to those with no preparation that could fall into a bias from their perception.

The evolution of technological tools around education is something that has come into academia in the past years; but from this study, it was clear that technology only plays a minimum role in the educational process in terms of teaching gender and sexual diversities. The role it has been playing is just as a medium for the real outcome, such as online surveys, online content, apps, or videos to complement the educational process, giving an area of opportunity for technological tools to be implemented and for ease of the educational processes. This means that the use of technological tools is also being excluded from studies around the topic.

Latin America, in general, is way behind the topic. The countries that consider the teaching or studying of sexual and gender diversities as part of their academia are very few, and none of the works considered for this review came from a Latin American country. Latin America has much work to do regarding the inclusion of people belonging to the LGBTQ+ communities. Year after year, the number of cases of violence within these countries is a sign of alarm, so the educational institutions could play an important role in the path of inclusion and understanding.

This study represents an analysis to understand the current position of the so-acclaimed inclusion within schools. The path to the creation of safe spaces for all types of people has been forthcoming for many years, and this study shows both the positive outcomes for those institutions that took the huge step and created curricula changes and all those sectors that are not mentioned in this study and that have been strangers to the path to inclusion. The inclusion of content regarding sexual and gender diversities is highly important to achieve the goals defined by the United Nations and thus improve the quality of life of people belonging to the LGBTQ+ community.

## Data availability statement

The raw data supporting the conclusions of this article will be made available by the authors, without undue reservation.

## Author contributions

Conceptualization and validation: CL-O and EL-C. Methodology, formal analysis, investigation, and data curation: CL-O. Writing—review and editing: CL-O, PP, and EL-C. Supervision: PP and EL-C. All authors contributed to the article and approved the submitted version.

## Conflict of interest

The authors declare that the research was conducted in the absence of any commercial or financial relationships that could be construed as a potential conflict of interest.

## Publisher's note

All claims expressed in this article are solely those of the authors and do not necessarily represent those of their affiliated organizations, or those of the publisher, the editors and the reviewers. Any product that may be evaluated in this article, or claim that may be made by its manufacturer, is not guaranteed or endorsed by the publisher.
